# A Hierarchical Bayesian Model to Predict Self-Thinning Line for Chinese Fir in Southern China

**DOI:** 10.1371/journal.pone.0139788

**Published:** 2015-10-06

**Authors:** Xiongqing Zhang, Jianguo Zhang, Aiguo Duan

**Affiliations:** 1 State Key Laboratory of Tree Genetics and Breeding, Key Laboratory of Tree Breeding and Cultivation of the State Forestry Administration, Research Institute of Forestry, Chinese Academy of Forestry, Beijing, 100091, P. R. China; 2 Collaborative Innovation Center of Sustainable Forestry in Southern China, Nanjing Forestry University, Nanjing, 210037, P. R. China; Southwest University, CHINA

## Abstract

Self-thinning is a dynamic equilibrium between forest growth and mortality at full site occupancy. Parameters of the self-thinning lines are often confounded by differences across various stand and site conditions. For overcoming the problem of hierarchical and repeated measures, we used hierarchical Bayesian method to estimate the self-thinning line. The results showed that the self-thinning line for Chinese fir (*Cunninghamia lanceolata* (Lamb.)Hook.) plantations was not sensitive to the initial planting density. The uncertainty of model predictions was mostly due to within-subject variability. The simulation precision of hierarchical Bayesian method was better than that of stochastic frontier function (SFF). Hierarchical Bayesian method provided a reasonable explanation of the impact of other variables (site quality, soil type, aspect, etc.) on self-thinning line, which gave us the posterior distribution of parameters of self-thinning line. The research of self-thinning relationship could be benefit from the use of hierarchical Bayesian method.

## Introduction

The self-thinning rule describes the mortality related to competition among trees within even-aged stands with full site occupancy [1, 2]. The rule has been a topic of research and discussion for more than eighty years [3–5]. In forestry, the rule has been used to develop relative density indices [6, 7], construct stand density management diagrams [8, 9], and serve as a predictor of stand growth [10, 11].

The best known and most widely used for describing the self-thinning relationship are those of Reineke’s equation [12], and the so-called -3/2 power law of self-thinning [3]. In Reineke’s equation, it described the relationship between the number of trees per unit area and quadratic mean diameter at breast height in even-aged stands of full density, whereas it described the relationship between mean plant biomass (or volume) and numbers per unit area in Yoda *et al*. The self-thinning rules of Reineke and Yoda *et al*. are useful and widely used in forest growth and yield models to predict natural mortality [13]. The two rules are basically the same in that both are used to quantify a maximum stand density for a given stand size. They assume a fixed slope (-1.605 for Reineke, and -1.5 for Yoda et al.) between the logarithm of size and the logarithm of density regardless of species, age, and site quality in fully stocked stands [11, 14]. However, since the late 1980s, the debate has primarily focused on whether the slope of the self-thinning line is invariant [15–17]. The debate can be explained by the variety of statistical methods that has been used to analyze the species self-thinning line [18].

There are several statistical methods to estimate the two parameters of the self-thinning line, such as (1) placing a line by hand above an upper boundary of data points [3, 7], (2) fitting least squares regressions[19–21], (3) estimating parameters through quantile regression [22], principal components analysis and reduced major axis regression (RMA) [23, 24], and RMA method combined with jackknife estimation [25], (4) bisector regression approach [26], and (5) adopting stochastic frontier function to estimate the self-thinning line [27, 28]. Zhang *et al*. [29] compared several statistical methods for estimating self-thinning relationship and found that the stochastic frontier analysis had important strengths over other methods such as ordinarily least squares regression, quantile regression, and principal components analysis.

Generally, the data is distributed across many different sites including different planting densities are used by researchers for analyzing self-thinning lines of a species. Implicit in the use of the estimated methods talked above on these data is that many different sites are assumed to be identical in behavior of the self-thinning (the same intercept and slope). But the assumption may not always be valid from theoretical and practical applications. Therefore, mixed effects models would be used to overcome the problem of data from different sites. VanderSchaaf and Burkhart [30] introduced the mixed effects models to estimate the self-thinning lines. Although the mixed effects model performed better than other methods, it gave only a single estimate for parameters instead of giving information about the uncertainty in parameters [31].

Generally, hierarchical Bayesian model is the other method to efficiently describe complex datasets and evaluate the uncertainty in parameters. For ecological modeling, information on the uncertainty in parameter estimates and model predictions are essential [32] and a Bayesian approach provides such information [33, 34]. A Bayesian model generates full distributions, allowing random parameter values and giving a more complete assessment of predictive uncertainty [35]. In addition, work experiences, published references and data are very important for doing forest researches. By incorporating the prior distribution, the Bayesian approach is more beneficial for forest management and decision-making.

Chinese fir (*Cunninghamia lanceolata* (Lamb.) Hook.), a fast growing evergreen coniferous tree, is one of the most important tree species for timber production widely distributed in southern China [36]. The objective of the study was to estimate self-thinning line for Chinese fir using Yoda *et al*.’s -3/2 law with a hierarchical Bayesian method. In addition, the hierarchical Bayesian method was compared with stochastic frontier function for estimating self-thinning line.

## Data

Data available for this study were from 15 permanent plots of Chinese fir plantations located in Shaowu County (27.08°N, 114.72°E), Fujian province, in southern China, which has a subtropical maritime monsoon climate, were established in 1982. Mean annual precipitation and mean temperature of this site is 1768 mm and 17.7°C, respectively. And monthly mean temperature ranges from 6.8°C in January to 28°C in July. The soil type is red, with rich soil humus contents. A detailed description of the data can be found in Sun *et al*. [25]. Sun *et al*. [37] found that when the mortality exceeded 10%, the self-thinning of Chinese fir plots occurred. In this study, we first selected data from plots for which the mortality exceeds 10% for modeling self-thinning line. The data for three plots with a planting density of 2 m×3 m, one plot with a density of 2m×1.5m and one plot with a density of 2 m×1 m were excluded since their mortality rates did not exceed 10% (trees/ha). Applying the principle, the data from 15 plots were reduced to 10 plots for the study of self-thinning line. Stem volume (dm^3^) of Chinese fir was obtained through the experimental equation developed by Liu and Tong [38]. Summary statistics for the plots are presented in Table 1.

**Table 1:** Descriptive statistics of stand variables of Chinese fir stands. (*n* = 10 plots).

Variables	Mean	SD	Min.	Max.
Age (years)	21	3.6	14	26
Density (trees/ha)	4877	1687.2	1833	8650
Average stem volume (dm^3^)	120.3	63.1	32.8	251.7

## Methods

### Self-thinning line

Yoda *et al*. [3] put forward an equation between average biomass (*W*) and number of trees per unit area (*N*). White [39] reported that there is a close relationship between the stem volume (*V*) and biomass *W*. In forestry, for practical reasons, biomass has usually been replaced by stem volume [3, 40]. The equation is given by:

lnV=a+bln(N) (1)

where *V* is the average stem volume in dm^3^, *a* and *b* are parameters to be estimated.

### Bayes rule

Detailed of the Bayes rule can be found in Zhang *et al*. [41]. In the study, the relationship between average stem volume *V* and number of surviving trees *N* is given by a statistical model:

$$\ln V∼N(g(\ln N:a,b),\sigma^{2})$$ (2)

Where *g*(ln *N*:*a*,*b*) = *a*+*b*ln(*N*). So the Bayesian rule equation in the study can be expressed by

p(data|a,b)p(a,b)=p(a,b|data)p(data) (3)

where the data consist of triples (ln*V*, ln*N*) measured from plots. In the current study, *p*(*data*|*a*,*b*) is the likelihood implied by Eq 4:

$$p(data|a,b)=\prod_{j}\frac{1}{\sqrt{2\pi }\sigma }\exp\left(\frac{-{\left(\ln(V_{j})-g(\ln N_{j}:a,b)\right)}^{2}}{2\sigma^{2}}\right)$$ (4)

Where *V*_*j*_ is the average stem volume of *j*th stand.

### Hierarchical Bayes

To determine if planting density affects the estimated values of the self-thinning line, we chose three models with different cluster-specific parameters for evaluation. They were varying intercepts without varying slopes (M1), varying slopes without varying intercepts (M2), and varying intercepts and slopes (M3), which listed respectively as:

$$\text{M}1:\;\ln V=(a+u_{0i})+b\ln(N)+\varepsilon$$ (5)

$$\text{M}2:\;\ln V=a+(b+u_{1i})\ln(N)+\varepsilon$$ (6)

$$\text{M}3:\;\ln V=(a+u_{0i})+(b+u_{1i})\ln(N)+\varepsilon$$ (7)

where *u*_0*i*_, *u*_1*i*_ are cluster-specific random effects to be predicted and assumed to be *N*(0, $\sigma_{0}^{2}$), and *N*(0, $\sigma_{1}^{2}$), respectively. A cluster is an individual plot (denoted by *i*). *ε* is assumed to be *N*(0, σ^2^). Variance $\sigma_{0}^{2}$ or $\sigma_{1}^{2}$ measures the between-subject variability, while σ^2^ accounts for the within-subject variability for all the plots.

### Prior distribution

The choice of prior distribution is critical for Bayesian method [42]. In the above model specifications, we need to choose appropriate prior distributions for all parameters. Non-informative normal (Gaussian) priors with large or infinite variance that reflect prior ‘ignorance’ are generally chosen. Alternatively, prior information can be available from the literature [41]. Based on the preliminary study, the prior of the parameter *b* was specified from the reported literature (Appendix A and B). All other parameters have non-informative priors (Table 2).

**Table 2:** Prior distributions of each parameter in M1-M3.

Parameter	Prior distribution
*b*	*b* ~*N*(-1.52, 0.39)
*a*	*a* ~*N*(0, 1000)
*σ*_0_	${\left(\frac{1}{\sigma_{0}}\right)}^{{}^{2}}∼Gamma(0.001,0.001)$
*σ*_1_	${\left(\frac{1}{\sigma_{1}}\right)}^{{}^{2}}∼Gamma(0.001,0.001)$
*σ*	${\left(\frac{1}{\sigma }\right)}^{{}^{2}}∼Gamma(0.001,0.001)$

### Model evaluation

In addition to the parameter estimates, the root mean square error (RMSE) and Deviance Information Criterion (DIC) were calculated for model performance evaluation.

$$RMSE=\sqrt{\sum_{j=1}^{n}{\left(y_{j}-{\hat{y}}_{j}\right)}^{2}/(n-1)}$$ (8)

Where *y*_*j*_ is observed volume of stand *j*, and ${\hat{y}}_{j}$ is the corresponding predicted value.

DIC is very useful in the Bayesian model selection [43], which is given by:

DIC=Dbar+pD (9)

where Dbar refers to the posterior mean of the deviance and pD is the effective number of parameters in the model. The posterior mean of the deviance Dbar = *E*_*θ*_(-2log(*p*(*DBH*|*θ*))), and pD = Dbar − Dhat. Dhat is a point estimate of deviance given by $\text{Dhat}=\text{-}2\log(p(DBH|\bar{\theta }))$. The model with the smallest DIC is selected to the “best” model.

The Bayesian method was implemented using the WinBUGS [44], which implements Markov chain Monte Carlo algorithms using a Gibbs sampler [45]. We used the R package R2WinBUGS [46] to link R and WinBUGS for data input and graph generation. In addition, we set 250 000 iterations to run to ensure the obtainment of maximum convergence and satisfied posterior distributions of estimated parameters. Among those 250 000 iterations, the initial 50 000 iterations were discarded from analysis as burn-in iterations. To reduce the correlation between neighbouring iterations, the thinning parameters were all set to 3.

In addition, we also used stochastic frontier function (SFF) described by Bi *et al*. [27] and Bi [47] to model the self-thinning line for comparing with the hierarchical Bayesian method. Details of this method were discussed by Bi *et al*. [27] and Bi [47]. The method was performed using LIMDEP 7 [48].

## Results

**Fig 1:**
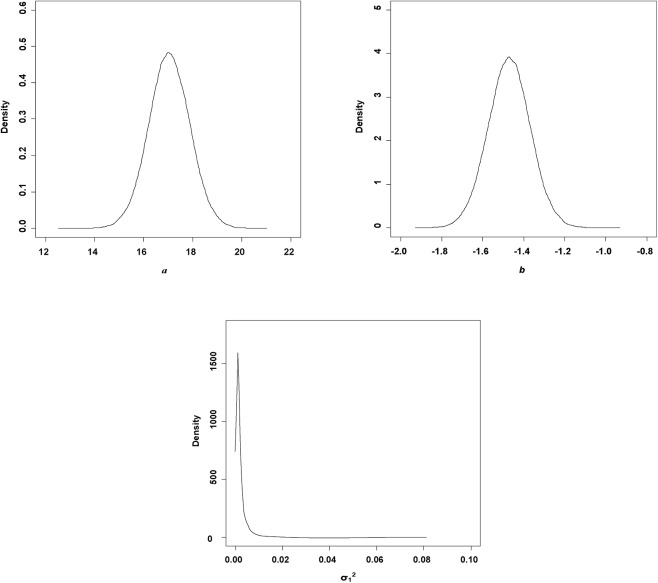
Hierarchical Bayesian estimates for the parameters in M2.

Comparing the parameter estimates from hierarchical Bayesian method with varying intercept without varying slope and varying slope without varying intercept, we found that they were quite close for the estimates of intercept and slope (Table 3). We also found that the parameter estimates from SFF method were similar with those of hierarchical Bayesian method (Tables 3 and 4). However, there was uncertainty existed in the self-thinning relationship, which could be showed by the posterior probability distributions of parameter estimates (Fig 1).

**Table 3:** Parameter estimates of self-thinning line relationships using hierarchical Bayesian method.

Model		Parameter estimate			95% interval		DIC	RMSE
		Mean	SD	Median	Lower	Higher		
M1	*A*	17.03	0.75	17.02	15.56	18.51	-27.0	0.1668
	*B*	-1.47	0.09	-1.47	-1.65	-1.29		
	$\sigma_{0}^{2}$	0.026	0.144	0.009	0.001	0.132		
	*σ* ^2^	0.032	0.007	0.031	0.021	0.048		
M2	*A*	17.04	0.84	17.04	15.38	18.70	-26.7	0.1657
	*b*	-1.47	0.10	-1.47	-1.68	-1.27		
	$\sigma_{1}^{2}$	0.002	0.007	0.001	0.0003	0.0114		
	*σ* ^2^	0.032	0.007	0.031	0.020	0.048		

**Table 4:** Parameter estimates of self-thinning line relationships using SFF method.

Parameter	Mean	SD	RMSE
*a*	17.04	0.62	0.1864
*b*	-1.47	0.07	

In addition, the random effects ($\sigma_{0}^{2}$, $\sigma_{1}^{2}$) were not significant both in intercept (mean = 0.026, SD = 0.144) of model M1 and slope (mean = 0.002, SD = 0.007) of model M2 of self-thinning line for Chinese fir (Table 3). Thus we did not compare the model M3 with M1 and M2. It indicated that both intercept and slope of self-thinning line are invariant regardless of initial planting densities. The posterior median of the total variation was found equal to 0.031 in model M1, from which 0.009 was attributed to between-subject variability. Correlation between measurements was relatively low (0.2903), which could be interpreted that only 29.03% of the total variation was due to between-subject variability, and 70.97% variance due to within-subject variability. This could also be found in model M2 that only 3.22% variance was due to between-subject variability, indicating that random effects do not improve the self-thinning model (Table 3).

**Fig 2:**
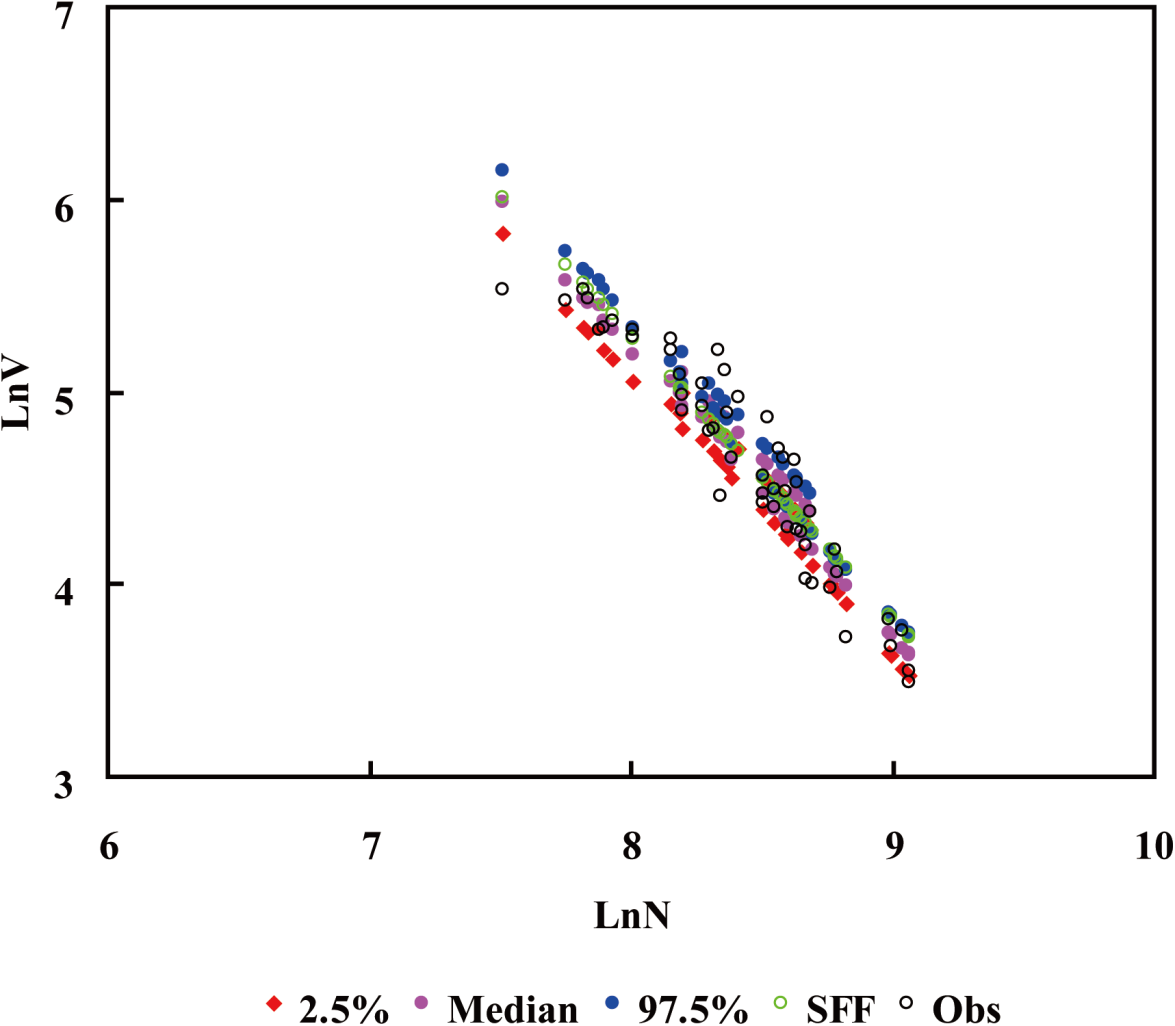
Scatter plots of predicted Ln-mean-volumes based on model M2 using hierarchical Bayesian method, and SFF method. Values of 2.5%, median, and 97.5% were obtained with hierarchical Bayesian method.

The hierarchical Bayesian method was better than SFF method according to the RMSE values (Tables 3 and 4), which could also be found in the fitted plots (Fig 2). In addition, model M2 was slightly better than M1 based on DIC and RMSE (Table 3).

## Discussion

One of the main debates on self-thinning rule has been the assumption of a constant slope regardless of planting density, age, and site quality [49, 50]. Instead of being a constant, studies reported that the slope of the self-thinning line was invariant across planting densities [30, 51]. Our study showed that the slope of self-thinning line remained constant across planting densities. The result of the work supports the argument of Puettmann *et al*. [49] and Tang *et al*. [50] who have concluded that the self-thinning line was invariant to planting density. Deng et al. [52, 53] also found that the slope of the self-thinning line remained constant in the crop plants. For the intercept of self-thinning line, it varies with species, and is a species-specific constant [3]. In this study, the random effect of planting density on intercept was not significantly different under four planting densities. Our findings disagree from those of others who have concluded that the intercept of self-thinning line was variable across planting densities [18, 54]. Although the random effect of planting density did not improve the model, we found the uncertainty of model predictions was mostly due to within subject variance.

Bi [46] and Pittman and Turnblom [55] found that site quality was a key factor influencing the self-thinning line in Douglas-fir (*Pseudotsuga menziesii*) and radiata pine (*Pinus radiata*), respectively. Zeide [56] reported that the effects of tree age and environment change made a 16% increase in the number of trees of a given size per unit as a result of changes in climate conditions. Harrington [57] and Weiskittel *et al*. [18] showed that the self-thinning line of red alder (*Alnus rubra*) was sensitive to aspect, moisture availability and dryness. So, there must be some uncertainty about the self-thinning relationship, which could be reflected by the posterior distributions of parameters of self-thinning line (Fig 1).

Over the last several decades researchers have applied different methods for analyzing self-thinning relationships. However, some of them have multiple limitations. Zhang *et al*. [29] reported that hand fitting method was an arbitrary and subjective technique. The estimated boundary line by quantile regression [22, 58] can be variable even with a small change in i^th^ quantile, and the variability is particularly high when sample used for analysis is small [29, 57, 59]. Mohler *et al*. [60] introduced use of principal comments analysis to estimate the self-thinning line. However, the method defines a “mean” self-thinning line [61] rather than a “real” self-thinning line [62] and does not provide an adequate calculation of the standard error associated with the parameters [24]. Bi *et al*. [27] and Bi [28, 47] adopted a stochastic frontier function to model the self-thinning line for even-aged pure pine stands, and provided an efficient estimation of the self-thinning upper boundary. However, the method ignores the parameters varying with sizes, which makes testing the influence of stand factor and site factors on the self-thinning relationship problematic [51]. Hierarchical Bayesian models provide a way forward by allowing for the uncertainty that cannot be assigned to specific causes. They are not restricted to individual effects, but also apply when uncertainty is structured in time, in space, and among different groups [63, 64].

In this study, we introduced hierarchical Bayesian method to estimate the self-thinning line. One of the advantages put forth in support of the hierarchical Bayesian model is the ability to incorporate prior information, such as from prior analyses, biomechanical argument, or expert opinion. For the analysis of self-thinning line we used an informative prior slope (*b*~*N*(-1.52, 0.39)) that comes from the published literature. The posterior distribution of the slope was shown to have a posterior mean of -1.47 suggesting that the self-thinning rule in Chinese fir plantations was similar with Yoda [3] rule -1.5 (Table 3).

Although Bayesian methods have been adopted in several applications in forestry [65–66], we are unaware of applications of hierarchical Bayesian techniques in parameterizing the self-thinning relationship. A number of slopes of self-thinning lines across different species gathered from published literature revealed that the slope can be described by a normal distribution. The normal distribution (Table 2) was used as a prior distribution in Bayesian framework in this study. It is the strengtheness of Bayesian method to update a model with prior distributions.

It should be noted additional variables can be included in the analysis that can obtain more accurate priors for new data for improving the research. For example, it would be possible to use the prior information adapting to soil type, and silvicultural management level. We also should note that the slope of self-thinning line may deviate from the idealized value as a result of unperfected circles of tree canopies at different stages [67]. In addition, the repeated measurement data may have the case of autocorrelation with time. If we take into account the autocorrelation under hierarchical Bayesian method, we believe that the research of self-thinning relationship could be benefit from further explorations of the use of Bayesian method.

## Supporting Information

S1 Table Estimated values of parameter *b* in self-thinning line collected from published literature. (DOC)

S1 Text Published literature estimating self-thinning line. (DOC)
