# Supplementary material for: A Hierarchical Bayesian Model to Predict Self-Thinning Line for Chinese Fir in Southern China
Source: PLoS One. 2015 Oct 6;10(10):e0139788. doi: 10.1371/journal.pone.0139788 (PMC4594911; doi:10.1371/journal.pone.0139788)
Supplement: S1 Table — (DOC) [file pone.0139788.s001.doc]

**S1 Table. Estimated values of parameter *b* in self-thinning line collected**

**from published literature**

| Estimate | Species | Reference |
| --- | --- | --- |
| -1.72 | *Pinus tabulaeformis* | 1 |
| -1.79 | *Larix pricipis-rupprechitii* | 1 |
| -1.37 | *Tsuga canadensis*-*Picea rubens* | 2 |
| -1.41 | *Abies balsamea*-*Picea* | 2 |
| -1.57 | *Thuja occidentalis*-*Picea mariana* | 2 |
| -1.475 | *Fagus sylvatica* | 3 |
| -1.485 | *Fagus sylvatica* | 3 |
| -1.472 | *Fagus sylvatica* | 3 |
| -1.374 | *Fagus sylvatica* | 3 |
| -1.49 | *Fagus sylvatica* | 3 |
| -1.595 | *Picea abies* | 3 |
| -1.648 | *Picea abies* | 3 |
| -1.614 | *Picea abies* | 3 |
| -1.594 | *Picea abies* | 3 |
| -1.633 | *Picea abies* | 3 |
| -1.624 | *Picea abies* | 3 |
| -1.467 | *Pinus sylvestris* | 3 |
| -1.369 | *Pinus sylvestris* | 3 |
| -1.667 | *Pinus sylvestris* | 3 |
| -1.392 | *Pinus sylvestris* | 3 |
| -1.617 | *Quercus petraea* | 3 |
| -1.847 | *Quercus petraea* | 3 |
| -2.139 | *Quercus petraea* | 3 |
| -1.41 | Pinus densiflora | 4 |
| -1.461 | *Abies balsamea* | 5 |
| -1.534 | *Abies balsamea* | 5 |
| -1.627 | *Abies balsamea* | 5 |
| -1.676 | *Abies balsamea* | 5 |
| -1.441 | *Abies balsamea* | 5 |
| -1.552 | *Picea mariana* | 6 |
| -1.214 | *Pinus banksiana* | 6 |
| -1.562 | *Picea mariana* | 6 |
| -2.1117 | *Abies fabri* | 7 |
| -1.938 | *Pinus massoniana* | 7 |
| -1.7102 | *Pinus armandii Franch.* | 7 |
| -2.5385 | *Cunninghamia lanceolata* | 7 |
| -1.9262 | *Picea aspoerata* | 7 |
| -1.553 | *Cunninghamia lanceolata* | 8 |
