# Supplementary material for: A Hierarchical Bayesian Model to Predict Self-Thinning Line for Chinese Fir in Southern China
Source: PLoS One. 2015 Oct 6;10(10):e0139788. doi: 10.1371/journal.pone.0139788 (PMC4594911; doi:10.1371/journal.pone.0139788)
Supplement: S1 Text — (DOC) [file pone.0139788.s002.doc]

**S2 Text. Published literature estimating self-thinning line**

1. Xue L, Ogawa K, Hagihara A, Liang S, Bai J. Self-thinning exponents based on the

allometric model in Chinese pine (*Pinus tabulaeformis* Carr.) and Prince Rupprecht’s larch

(*Larix principis-rupprechtii* Mayr) stands. For Ecol Manage. 1999; 117: 87-93.

2. Solomon D, Zhang L. Maximum size-density relationship for mixed softwoods in the

northeastern USA. For Ecol Manage. 2002; 155: 163-170.

3. Pretzsch H. Species-specific allometric scalling under self-thinning: evidence from

long-term plots in forest stands. Oecologia 2006; 146: 572-583.

4. Xue L, Hagihara A. Self-thinning lines of organs and aboveground parts based on

allometric relationships in overcrowded *Pinus densiflora* stands. Ecol Res. 2012; 1: 15-21.

5. Bégin E, Bégin J, Bélanger L, Rivest LP, Tremblay S. Balsam fir self-thinning relationship

and its constancy among different ecological regions. Can J For Res. 2001; 31: 950-959.

6. Newton PF. Asymptotic size-density relationships within self-thinning black spruce and

jack pine stand-types: Parameter estimation and model reformulations. For Ecol Manage.

2006; 226: 49-59.

7. Liu J. The role of *a* of Maximum-size line V=KNm^-a^ about main timber forest in south of

China. Central South Forest Inventory and Planning 1989; 3: 30-34. (in Chinese)

8. Sun H, Zhang J, Duan A, He C. Estimation of the self-thinning boundary line within

even-aged Chinese fir (*Cunninghamia lanceolata* (Lamb.) Hook.) stands: Onset of

self-thinning. For Ecol Manage. 2011; 6: 1010-1015.
